# Translation factors that keep the ribosome Pro-active

**DOI:** 10.1128/mmbr.00017-26

**Published:** 2026-08-06

**Authors:** Heather A. Feaga

**Affiliations:** 1 Department of Microbiology, Cornell University 251789, Ithaca, New York, USA; National Cancer Institute, Bethesda, Maryland, USA

**Keywords:** ribosome, translation, bacteria

## Abstract

Ribosomes produce the staggering array of proteins that perform the structural and enzymatic feats of the cell. Therefore, most of the cell’s energy goes toward producing ribosomes and the work performed by them. The work of the ribosome is relatively simple—decode mRNA codons and catalyze the formation of peptide bonds. By marching iteratively along the length of an open reading frame, a complete peptide is produced. Ribosomes catalyze the formation of peptide bonds at a rate of approximately 15 amino acids per second. However, bonds between amino acids do not form with equal efficiency, and ribosomes can become stalled at difficult-to-translate sequences. Proline is unique among the amino acids in that its side chain is covalently bonded to the peptide backbone to form a rigid ring. The rigidity of proline, and especially tracts of proline, makes it a difficult substrate for peptide bond formation, but it is also an essential motif in many protein structures. Elongation factor P (EF-P) is the star player for facilitating translation of polyproline tracts. However, recently identified factors play an important supporting role, and loss of these factors incurs a severe fitness defect in the absence of EF-P. These factors include an EF-P paralog, EfpL, as well as the ABCF ATPase YfmR/Uup and YebC2. The abundance and partial redundancy of factors that prevent ribosome stalling at polyprolines highlights the structural importance of polyproline tracts and the need to facilitate their translation. Here, we review recently identified translation factors that prevent ribosome stalling at polyprolines in bacteria.

## INTRODUCTION: THE STEPS OF TRANSLATION

**Fig 1:**
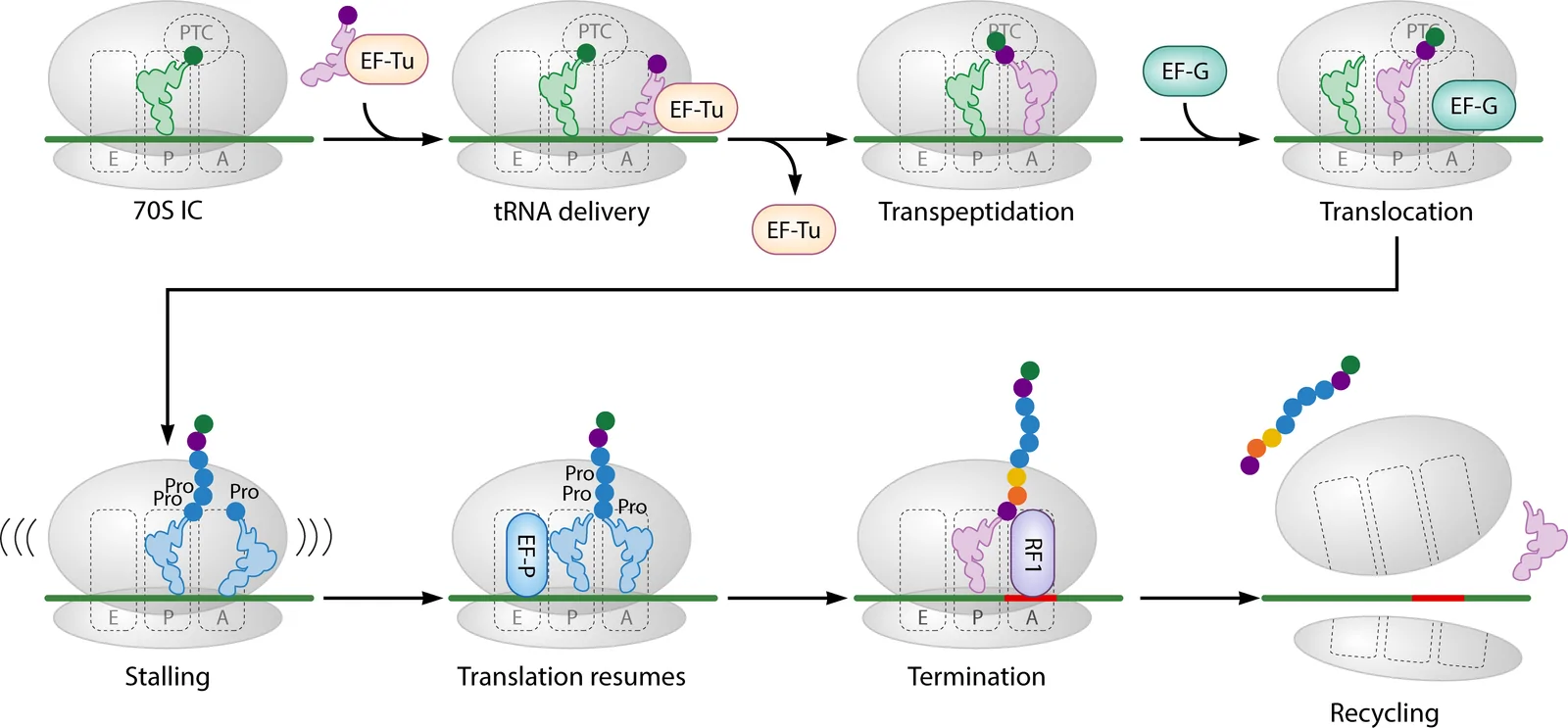
Overview of the steps of translation. Translation initiates at a start codon. EF-Tu delivers charged tRNAs to the A site of the ribosome. If a cognate tRNA is delivered, accommodation will occur, and EF-Tu will exit the ribosome. The large subunit of the ribosome catalyzes peptide bond formation at the peptidyl transferase center (PTC); EF-G then translocates the mRNA by one codon. If the ribosome becomes stalled at a polyproline tract, EF-P binds between the P and E sites and stabilizes the peptidyl-tRNA to facilitate translation. When a stop codon is reached, a class 1 release factor (RF1 or RF2) will terminate translation, and the large and small subunits will be recycled by the combined action of EF-G and RRF.

Translation begins with the binding of mRNA to the small subunit of the ribosome (1, 2) (Fig. 1). The start codon is typically selected based on its sequence context. For example, most bacteria rely on a Shine-Dalgarno sequence positioned approximately 6–15 nucleotides upstream of the start codon (3, 4). The Shine-Dalgarno sequence is purine-rich and base-pairs with the anti-Shine-Dalgarno sequence at the 3′ end of the 16S ribosomal RNA of the small subunit. An initiator tRNA (tRNA^fMet^) bound to IF2 is then positioned at the P site to initiate translation. The large subunit joins, IF2 exits, and the ribosome is then ready to proceed with translation elongation.

After initiation, all subsequent tRNAs are delivered by EF-Tu to the A site of the ribosome. Each incoming tRNA is charged with an amino acid at its 3′ end and uses its anticodon to base-pair with the mRNA codon. Correct base pairing triggers a conformational change in the ribosome, which allows accommodation of the charged stem of the tRNA into the peptidyl transferase center (PTC) and release of EF-Tu (5, 6). Here, in the PTC, the peptide bond is formed between the amino acid attached to the P-site tRNA and the incoming amino acid attached to the A-site tRNA (7). The nascent polypeptide is transferred from the P-site tRNA and becomes covalently linked to the A-site tRNA. Then, through ratcheting of the small subunit of the ribosome and the action of EF-G, the A- and P-site tRNAs and the mRNA are translocated by one codon, with the former A-site tRNA now occupying the P site (8–12). After translocation, the next codon is positioned in the A site, ready to be decoded by the next tRNA. This process continues at a rate of approximately 15 codons per second until a stop codon is reached (13, 14).

When the ribosome reaches a stop codon, a release factor catalyzes hydrolysis of the ester bond between the peptide and the P-site tRNA. RF1 terminates translation at UAA and UAG stop codons, whereas RF2 terminates at UAA and UGA stop codons (15). Hydrolysis of the ester bond linking the peptide to the tRNA is similar to the transpeptidation reaction, except that the participating nucleophile is a water molecule rather than the alpha amine of the incoming amino acid. Both RF1 and RF2 rely on a GGQ motif, which they share with the class I release factor of eukaryotes (eRF1), even though eRF1 evolved independently. The GGQ motif has long been hypothesized to directly coordinate a water molecule for nucleophilic attack on the carbonyl carbon of the last amino acid in the peptide chain (16). Recently, this mechanism was resolved further. Release factors cause a puckering of the terminal adenosine (A76) of the peptidyl-tRNA such that its 2′ OH attacks the adjacent carbonyl carbon, making it accessible to further nucleophilic attack by a water molecule that then hydrolyzes the peptide from the peptidyl-tRNA (17). Such a mechanism is supported by the spontaneous self-hydrolyzing property of RNA, which similarly involves nucleophilic attack by the 2′ OH of ribose on the phosphate backbone of RNA (18).

## PROLINE IS UNIQUE AMONG THE AMINO ACIDS

**Fig 2:**
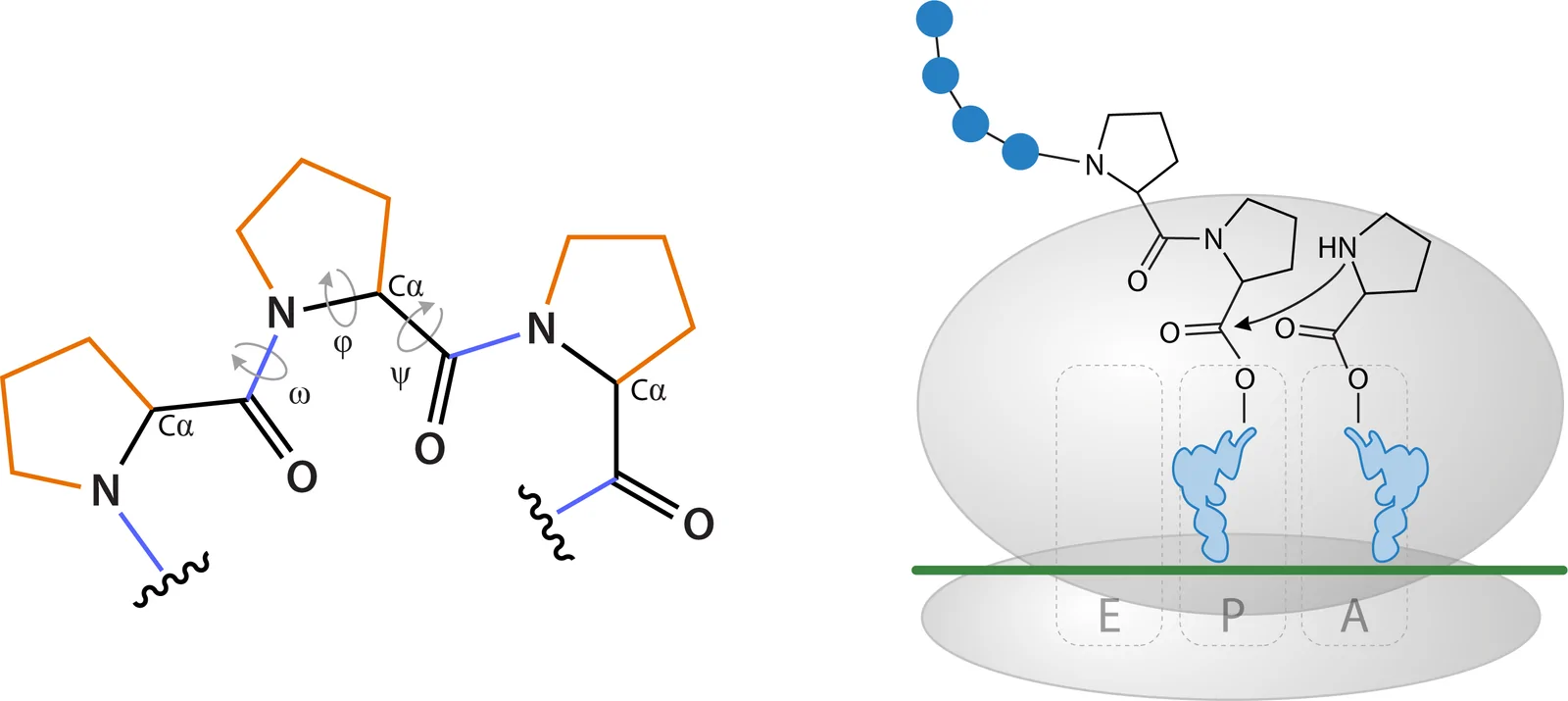
Proline possesses a unique geometry that makes polyproline tracts difficult for the ribosome to translate. (Left) Three consecutive prolines in the *trans-*conformation. The peptide bond is colored blue. The side chain is colored orange. Phi, psi, and omega angles of rotation are indicated. (Right) Schematic of peptide bond formation on the ribosome. The alpha amine of proline in the A site performs a nucleophilic attack on the carbonyl carbon of the proline attached to the P-site tRNA.

There are 20 standard proteinogenic amino acids in nature. Among the proteinogenic amino acids, proline is the only amino acid in which the side chain circularizes and is covalently bound to the peptide backbone. This unique structure imparts several important properties to proline. First, proline is more rigid than the other amino acids since the phi angle of the peptide backbone is restricted (19) (Fig. 2). Second, the connection of the side chain to the nitrogen of the alpha amine eliminates a critical hydrogen that could participate in hydrogen bonding in an alpha helix or beta sheet. Both the rigidity of proline and the absence of hydrogen on the alpha amine contribute to proline’s function as a disruptor of alpha helices and beta sheets (20). The connection of the alkyl group to the nitrogen in proline also decreases the nucleophilicity of proline because its electron pair is poorly positioned for nucleophilic attack. Therefore, of all the proteinogenic amino acids, it is most difficult for proline to form a peptide bond.

Proline is also unique in that its side chain can assume both *cis*- and *trans*-conformations with respect to the peptide backbone with near equal efficiency. All other amino acids nearly always assume a *trans*-conformation to avoid steric clash with neighboring side chains (21). For proline, while the two conformations are nearly isoenergetic, isomerization between the conformations is a slow process and is often facilitated by prolyl isomerases, such as Pin1 in eukaryotes (22). Proline isomerization between *cis*- and *trans*-conformations is a major regulator of the activity of many proteins and can serve as a powerful molecular switch to regulate cellular processes, including gene expression, cell cycle progression, and the immune response (23). For notable examples of protein regulation by proline isomerization, see P53 (24, 25), histone H3 (26), JNK (27), NFkB (28), membrane transporters (29), and RNA Polymerase II (30). The unique structure of proline residues also helps them form their own specialized helices. Polyprolines in a left-handed *trans*-conformation form the PPII-helix that is ubiquitous in the eukaryotic proteome (31). First thought to be mainly important in fibrous proteins like collagen and titin, PPII-helices are now known to play roles in globular proteins (32). Moreover, proline-rich proteins have major roles in modulation of the immune response and cell signaling (33).

There is a high degree of variability in both the length and number of polyproline tracts in various organisms. The human proteome contains 10,000 polyproline motifs with 3 or more consecutive prolines, and some proteins have as many as 27 consecutive prolines (34, 35). Bacteria are restricted in the number of consecutive prolines tolerated within a coding sequence. For example, the model *Escherichia coli* strain MG1655 encodes 2,077 diproline motifs, 102 triproline motifs, and a maximum of 8 consecutive prolines in *amiB,* which encodes a cell wall amidase that contributes to cell division (36). The maximum number of consecutive prolines encoded in the *Bacillus subtilis* genome is four, suggesting that *B. subtilis* has a lesser tolerance for translating long polyproline stretches. Meanwhile, *Mycobacterium tuberculosis* is exceptional in the number of polyproline motifs, due in large part to the PPE family proteins that are defined by conserved proline and glutamate residues near the N-terminus. PPE proteins make up approximately 10% of the *M. tuberculosis* genome, but their biological roles are largely mysterious (37, 38). They are typically membrane localized or secreted and may be involved in virulence, adherence, and nutrient uptake (37, 38).

**Fig 3:**
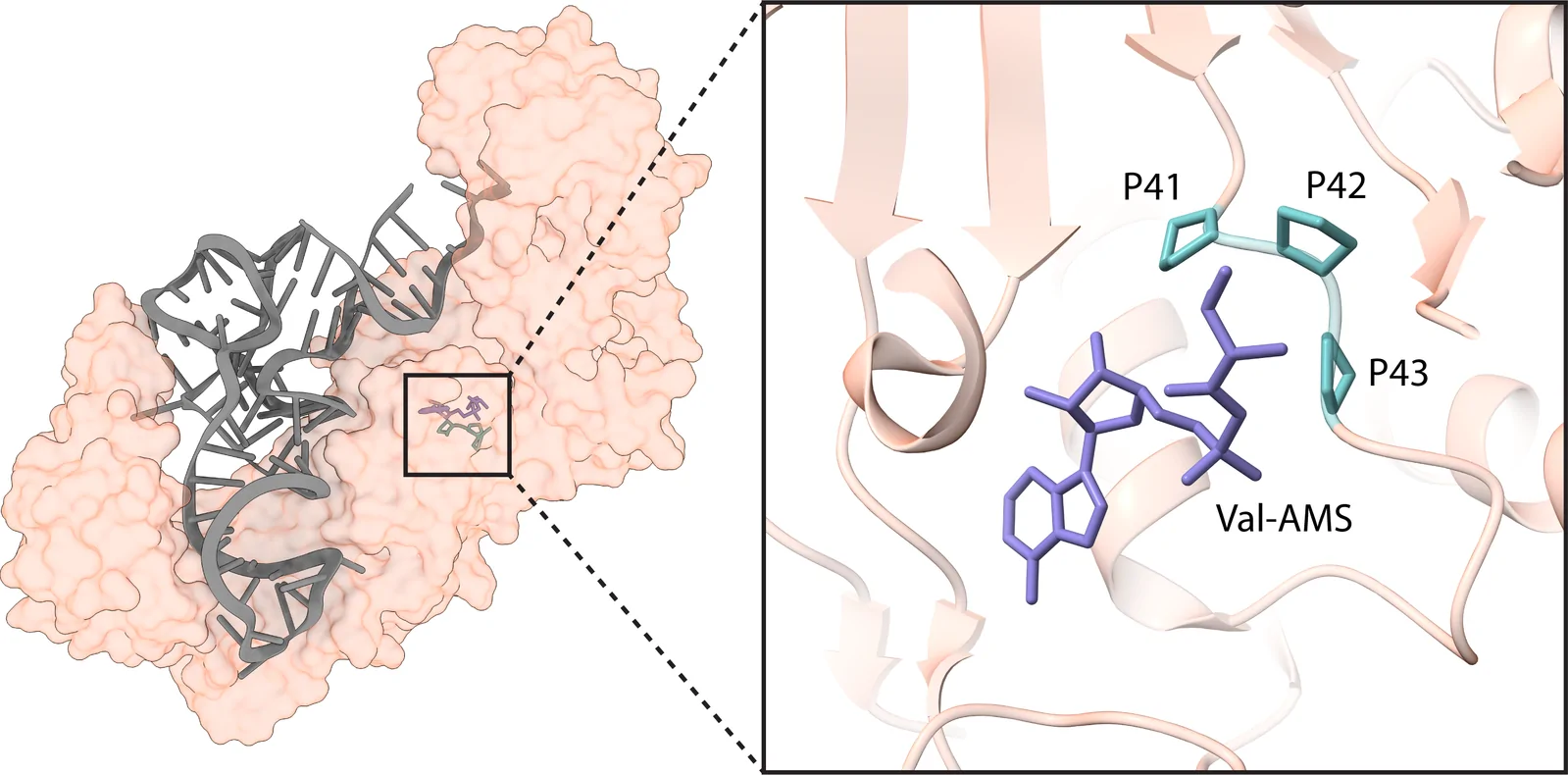
The conserved tri-proline motif in ValS. The valine tRNA synthetase ValS contains a tri-proline motif in the active site where Val-AMS is formed from valine and ATP prior to tRNA charging. Modeled with PDB 1GAX.

Polyproline tracts also occur in some essential genes, with the most notable example being *valS*, which encodes the valine tRNA synthetase. ValS is conserved in all organisms, and almost all orthologs encode a PPP motif that is important for its function (39, 40) (Fig. 3). Exceptions to this rule are found within Planctomycetota, where ValS contains PLP in place of the universally conserved proline triplet (41). It appears that for these species, an ancestor lost its native copy of *efp* and received a non-native *efp* by horizontal gene transfer; the gain of a non-optimal EF-P likely selected for mutation of the proline triplet in ValS (41). The bacterial gene with the next most highly conserved polyproline motif encodes another tRNA synthetase, IleS. IleS contains a GPP motif in the same position as the PPP motif in ValS in the active site. IleS is essential, but stalling at GPP in *ileS* in the absence of EF-P is not as severe as the ribosome stalling that occurs at the PPP motif in *valS* (42).

## POLYPROLINE TRACTS ARE DIFFICULT TO TRANSLATE AND CAUSE RIBOSOME STALLING

During peptide bond formation, the alpha-amine of the A site amino acid performs a nucleophilic attack on the carbonyl carbon of the amino acid in the P site. *In vitro* assays monitoring dipeptide synthesis reveal that when tRNA^fMet^ is in the P site, and proline is the incoming amino acid, the reaction proceeds normally, forming with similar efficiency as any other amino acid (43). However, when proline is the amino acid in both the A site and the P site, this reaction proceeds slowly (44, 45). A poor nucleophile even in solution (44), proline is even more poorly positioned to perform the nucleophilic attack in the context of the ribosome PTC (35, 46–48). Typically, the side chain of the incoming amino acid is cradled in the A-site cleft of the PTC (49, 50). For proline, the side chain ring is not positioned properly in the cleft, which results in a poor geometry for peptide bond formation (35). Consecutive prolines in the nascent chain also destabilize the P-site tRNA, further interfering with positioning (51). The start of the exit tunnel itself likely poses an additional barrier to the synthesis of polyproline tracts. Peptide bonds are made in the *trans*-conformation (52, 53), and consecutive prolines in the all *trans-*conformation form a bend that may sterically clash with the entry to the exit tunnel (51). Altogether, these properties make proline both a poor donor and acceptor of the peptide bond, and the ribosome stalls.

Ribosome stalling is deleterious because it can result in the production of aberrant, truncated peptides and/or interfere with protein folding. Beyond these direct consequences, ribosome stalling also causes ribosome collisions, which occur when a trailing ribosome is elongating more quickly than the ribosome preceding it (54). In *E. coli*, ribosome collisions cause recruitment of SmrB, a nuclease that cleaves mRNA between the stalled and collided ribosome (55). mRNA cleavage results in a truncated message that is useless for further translation, and all trailing ribosomes will need to be rescued either by *trans*-translation or one of the alternative rescue pathways (56–58). The activity of *trans*-translation causes the resulting truncated proteins to be targeted for degradation (59). In *B. subtilis*, ribosome collisions recruit MutS2, which separates the large and small subunits of the stalled ribosome from the mRNA using its ATPase activity (60, 61). MutS2 contains an SMR domain that is homologous to the SMR domain of SmrB. However, the SMR domain of MutS2 does not possess nuclease activity, and so it does not cleave mRNA to trigger *trans*-translation-mediated rescue (61). Instead, its crucial function is ribosome splitting, which leaves a large ribosomal subunit that is obstructed by peptidyl-tRNA, which must be subsequently rescued by the activity of RqcH and PTH (62). The importance of mechanisms that rescue stalled ribosomes is highlighted by their conservation—*trans*-translation is conserved in >97% of bacterial genomes (58)—and by the deleterious effects of loss of these factors (57).

## EF-P STABILIZES PEPTIDYL-tRNA TO FACILITATE TRANSLATION

**Fig 4:**
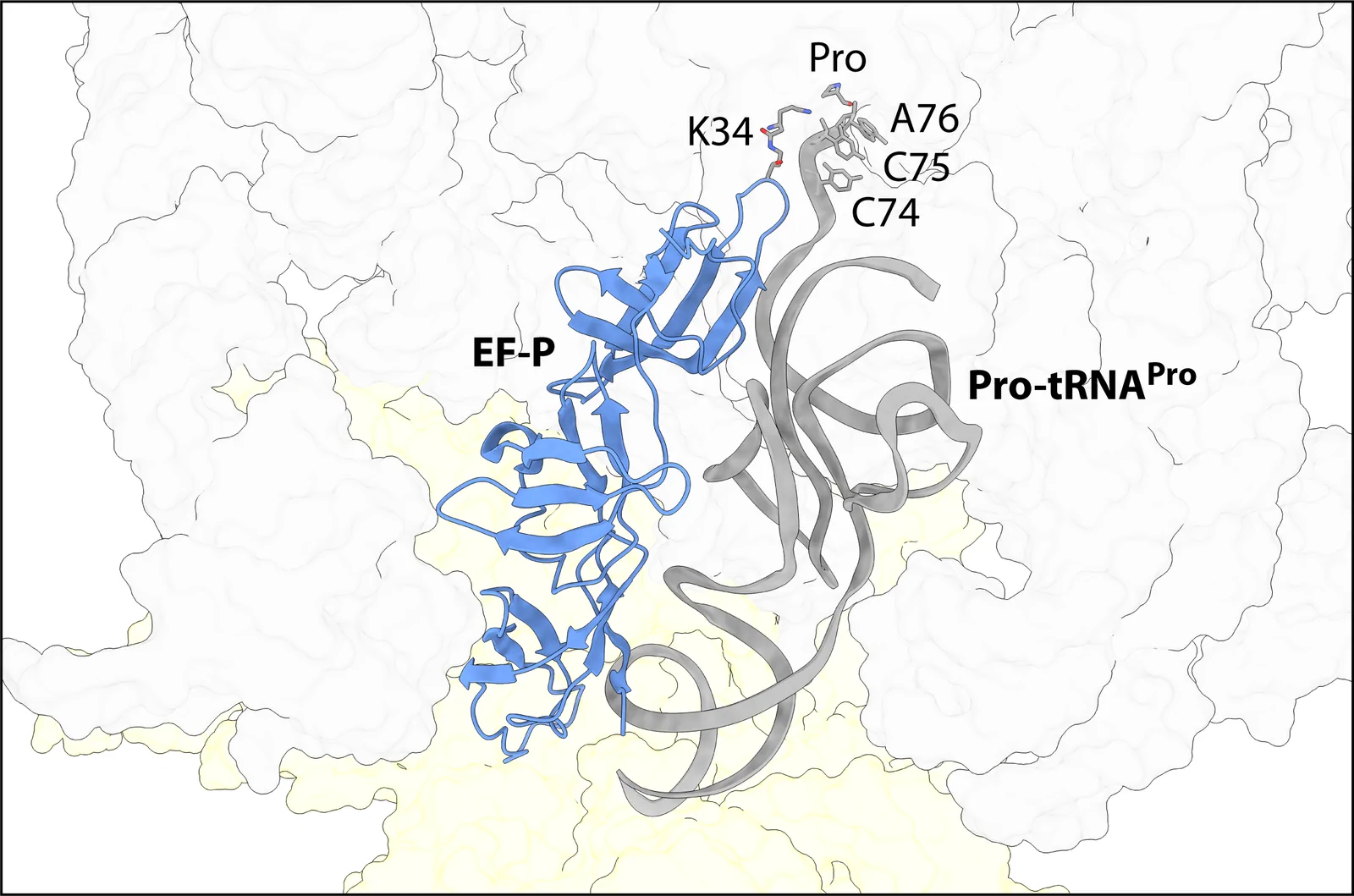
Model of EF-P bound to the elongating ribosome. EF-P binds the ribosome between the P and E sites to stabilize the peptidyl-tRNA and facilitate peptide bond formation. EF-P is shown in blue, and the P-site tRNA charged with proline is in gray. Lysine 34 is post-translationally modified and extends toward the PTC. Modifications vary depending on the species, and in some species, Arg takes the place of Lys. Modeled with PDB 6ENU.

EF-P is a specialized translation factor that relieves ribosome stalling at XPPX motifs (63, 64). It mimics the shape of a tRNA and binds to the ribosome between the P and E sites (65). EF-P is a three-domain protein. Domain I makes extensive contacts with the P-site tRNA, exhibiting some specificity for tRNA^fMet^ and tRNA^Pro^, while domain III makes weak contacts with the E-site codon (Fig. 4) (51, 66). Domain I extends toward the PTC, where it may alter the conformation of the P-site tRNA and nearby rRNA nucleotides to facilitate transpeptidation. In most bacteria, a conserved lysine or arginine residue located at the tip of domain I is post-translationally modified and contributes to EF-P’s function (67).

EF-P was originally identified as an extra-ribosomal factor that could stimulate bond formation between initiator f-Met and puromycin (68). This finding suggested that EF-P may act as a general elongation factor and led to its being named elongation factor peptidyl. Subsequent analysis of the stimulatory effect of EF-P on peptide bond formation revealed that it could promote peptide bond formation between initiating methionine and glycine or leucine, and, to a far lesser degree, between lysine, methionine, or phenylalanine (69). The first structure of EF-P was determined on an initiating ribosome with tRNA^fMet^ in the P site (70). While these biochemical and structural data indicated that EF-P may promote the first peptide bond, it was soon to be revealed that EF-P’s major role is in elongation.

In 2013, two papers published together in *Science* revealed the role of EF-P in promoting elongation, especially at XPPX motifs (45, 71, 72). Using a biochemical approach, the Rodnina lab showed that EF-P was absolutely required for correct synthesis of longer peptides such as fMPPG, fMPPGF, or fMPPPF (45). They also determined that EF-P prevented ribosome stalling at polyproline tracts during translation of full-length proteins, such as the AmiB protein of *E. coli* that contains eight consecutive prolines. Meanwhile, the Jung and Wilson labs took a genetic approach, using transposon mutagenesis to identify genes that were important for the expression of the membrane-associated Cad module that is important for acid resistance (71). Incidentally, a member of this module, CadC, contains a PPPIP motif that requires EF-P for efficient translation. These two studies solidified the role of EF-P in translation elongation and highlighted the utility of biochemical as well as genetic approaches for determining the role of translation factors.

Subsequent work indicates that EF-P is important for relieving ribosome stalling at polyproline tracts as well as other difficult-to-translate sequences (63, 73, 74). Ribosome profiling studies have been especially informative, revealing global positions of ribosome stalling across the transcriptome in the absence of EF-P (42, 75–79). These studies have confirmed that polyproline sequences are especially problematic, but that the residue immediately upstream or downstream of a di-prolyl motif also determines whether ribosomes will stall. For example, in *E. coli*, the PPX motifs with highest pause scores are consistently PPW, PPN, PPD, PPG, and PPP (42, 75, 76, 79). These studies have also given insight into which genes are likely to be poorly expressed in the absence of EF-P and can often explain phenotypes exhibited by ∆*efp* mutants. For example, the significant ribosome stalling signature at the SPP di-prolyl motif in the flagellar component FliY in *B. subtilis* is consistent with the lack of swarming motility exhibited by ∆*efp* cells (77). However, ribosome stalling detected by ribosome profiling does not automatically indicate that less protein will be produced. The strength of the stall, levels of translation initiation, and how close the stalling site is to the beginning of the open reading frame all determine whether protein output will be affected (76).

In 2017, the structure of EF-P bound to a polyproline tract on an elongating ribosome was determined by the Wilson lab (51) (Fig. 4). Importantly, these stalled ribosomes were in the process of translating a polyproline tract that was ~70 codons into the mRNA and therefore approximates a physiological substrate. In the absence of EF-P, the CCA stems of both P- and A-site tRNAs were poorly resolved, suggesting a lack of stabilization of both tRNAs. Moreover, the N-terminus of bL27 was disordered, which is consistent with the A-site tRNA failing to become accommodated (52, 80). When EF-P was added to the polyproline-stalled ribosomes, the N-terminus of bL27 became ordered, indicating that tRNA accommodation had occurred. After the addition of EF-P, most particles represented nascent chains bound to the A-site tRNA, indicating that EF-P had promoted transpeptidation. The addition of EF-P also stabilized the peptidyl-tRNA, as EF-P makes direct contacts with the CCA stem of this tRNA. Since peptide bonds are produced in the *trans*-conformation, and since an all-*trans*-conformation of a tri-proline motif would cause a steric clash with G2061 in the exit tunnel, it is thought that EF-P promotes an “alternative *trans*-conformation” of the peptide (51). An all-*trans* left-handed proline helix (PII helix) exhibits Phi and Psi angles of approximately −75° and 145°, respectively (32, 81). In the presence of EF-P, the Pro-Pro moiety of the nascent peptide adopts a slightly different Phi angle of −90° and Psi angle of 120°. This configuration of the peptide may promote its exit from the ribosome as well as a favorable geometry for peptide bond formation.

EF-P is universally conserved, with homologs present in eukaryotes (eIF5A) and archaea (aIF5A). In all eukaryotes that have been investigated, EF-P is essential (82–85), which may be due to its more general role in translation (86). Attempts to delete aIF5A have also been unsuccessful, suggesting essentiality in archaea (87). In bacteria, phenotypes vary greatly depending on the species. The gene encoding EF-P is essential in pathogenic bacteria, including *Mycobacterium tuberculosis* (88), *Neisseria meningitidis* (89)*,* and *Acinetobacter baumannii* (90), and in the Planctomycete *Planctopirus limnophilia* (91). For bacterial species in which EF-P is not essential, these organisms exhibit pleiotropic phenotypes, consistent with varied effects on translation. These phenotypes include decreased motility and sporulation (77, 78, 92), reduced antibiotic resistance (93, 94), defects in virulence (95), and reduced growth rate (96, 97).

## EF-P IS POST-TRANSLATIONALLY MODIFIED

EF-P is post-translationally modified in most bacteria. Post-translational modification occurs on the conserved lysine or arginine that are in equivalent positions at the tip of domain I. In *E. coli* and other Gammaproteobacteria, Lys34 undergoes (R)-beta-lysylation and an additional, but dispensable, hydroxylation (74, 98–101). Lys34 and its modification directly interact with the CCA stem of the P-site tRNA (51) (Fig. 4), and modification is essential for stable association of EF-P to the ribosome (66). Consistent with these important roles, loss of (R)-beta-lysylation in *E. coli* and *Salmonella enterica* greatly reduces the activity of EF-P *in vitro* and *in vivo* and negatively impacts growth and virulence (74, 94, 102).

Firmicutes exhibit 5′-aminopentanolation of the conserved lysine at the tip of domain I (Lys32) (103, 104). In *B. subtilis*, EF-P modification occurs through a multi-step process, catalyzed by GsaB, YnbB, and YmfI. The modification state variably impacts transpeptidation at PPP, PPE, and PPW motifs. Interestingly, in the case of swarming motility, partial EF-P modification is more detrimental than total loss of the modification (103, 104). Inactivation of YmfI (which causes Lys32 to be modified with 5-aminopentanone instead of 5-aminopentanol) severely reduces swarming motility, yet motility is significantly rescued by additional inactivation of YnbB, an enzyme that acts upstream of YmfI in the modification pathway. As discussed in a later section, double deletion of genes encoding both EF-P and the ATPase YfmR incurs a severe synthetic fitness defect in *B. subtilis* (105). However, there is no detectable growth defect incurred by double deletion of *yfmR* and the genes encoding GsaB, YnbB, or YmfI (105). Altogether, these data suggest that the post-translational modification of EF-P in *B. subtilis* is not essential for its activity.

Many Betaproteobacteria, such as the pathogens *Pseudomonas aeruginosa* and *Neisseria meningitidis*, encode arginine at the tip of domain I, which is rhamnosylated by EarP (106, 107). Deletion of either *efp* or *earP* from *P. aeruginosa* greatly reduces growth and motility and increases antibiotic susceptibility (93). EF-P, but not EarP, is essential in *N. meningitidis*; however, *earP* deletion incurs a severe growth defect (89). Therefore, in addition to EF-P, enzymes responsible for post-translational modification of EF-P are promising antibiotic targets.

## THE EF-P PARALOG EfpL

Many bacteria encode a paralog of EF-P, EfpL, which shares 30% sequence similarity with EF-P but forms a distinct phylogenetic branch (41). This paralog is widespread among Gammaproteobacteria and is also found in some Planctomycetota, Acidobacteria, and Desulfobacterota (41, 79). All species that encode EfpL also encode EF-P, suggesting that it plays a supporting role in translation (41). It may also not be as highly expressed as EF-P, since in *E. coli* there are 10-fold fewer EfpL proteins than EF-P (~4,500 vs ~40,000) (79, 108).

EfpL has a three-domain structure that is similar to EF-P, including the conserved loop region in domain I that interacts with the PTC (109). For EF-P, the tip of this domain contains either a conserved lysine or arginine that is typically post-translationally modified in bacteria and eukaryotes. EfpL encodes a conserved arginine at the tip of domain I, which has been confirmed by mass spectrometry to be unmodified (79). However, the loop region of domain I is extended by two amino acids, which could allow it to better reach the PTC in the absence of the post-translational modification. A similar loop extension (present in a subfamily called PGKGP type EF-Ps) is employed by Actinobacteriota that encode unmodified EF-P (110, 111). The lack of modification of EfpL may explain its reduced activity, but the precise function of EF-P modifications is not yet clear. Additional EF-P modifications are yet to be determined, as approximately half of bacterial genomes encode an EF-P type that does not fall into the known modification classes (41).

Ribo-seq experiments recently performed by the Lassak lab reveal robust, but not complete, overlap in EF-P vs EfpL substrates (79). Consistent with a probable supporting role in translation, single deletion of *efpL* caused ribosome stalling levels that are similar to wild type. However, overexpression of EfpL in ∆*efp* cells alleviates ribosome stalling at many stalling motifs, especially PPN, PPP, and PPG. Substrate overlap is also supported by genetic evidence, since ∆*efp*∆*efpL* cells have a growth defect that is more pronounced than in either single deletion, and overexpression of EfpL reduced the doubling time of ∆*efp*∆*efpL* cells below that of ∆*efp* (79). These data support the possibility that protein levels may explain the reduced activity of EfpL, rather than reduced functioning of the protein.

Ribosome profiling experiments show that the single ∆*efpL* deletion did not increase ribosome pausing scores relative to wild type (79). However, there were some differences in gene asymmetry scores between wild-type and ∆*efpL* cells. Asymmetry scores compare ribosome density in the first half of a gene to the latter half of the gene (112). Genes with a high degree of ribosome pausing will give a high asymmetry score since there will be greater ribosome occupancy prior to the stall site than after the stall site. Genes with greater asymmetry scores in ∆*efpL* cells included genes encoding amino acid metabolism and transport. Therefore, EfpL may play a greater role in low-nutrient environments. Interestingly, taxa that encode the EF-P paralog are predicted to have higher growth rates based on codon usage bias (79). This observation is consistent with a study from the Ibba lab that showed that *E. coli* grown at optimal temperatures demonstrate a stricter need for EF-P than *E. coli* growing more slowly at non-optimal temperatures (97). Faster growth would likely exert greater selective pressure on the speed of protein synthesis. Consistent with this prediction, slow-growing thermophilic bacteria are enriched in polyproline motifs compared to fast-growing mesophiles, which have limited the number of polyproline tracts in their genomes (41).

## ABCF ATPases PROMOTE TRANSLATION OF PROLINE-RICH AND CHARGED TRACTS

Variability in the severity of phenotypes upon *efp* deletion suggests that there are alternative factors for facilitating polyproline translation across bacterial species. To search for these factors, Hong and colleagues (113) performed a Tn-seq screen to identify genes whose disruption incurs reduced fitness in ∆*efp* cells and may therefore indicate a redundant function. This approach led to the identification of YfmR (an ABCF ATPase) as a ribosome-associated factor that facilitates translation of polyproline tracts. Meanwhile, Takada and colleagues (105) noted that ABCF ATPases possess a domain that interacts with the PTC, which is referred to as the peptidyl-tRNA interacting motif (PtIM). Therefore, they correctly hypothesized that some ABCFs could relieve ribosome stalling at specific sequences and found that YfmR could reduce ribosome stalling at proline-rich sequences, and the ABCF YkpA could resolve stalling at highly charged tracts of amino acids. At the same time, Chadani and colleagues (114) were the first to show that an ABCF can alleviate ribosome stalling at a polyproline tract *in vitro*. They found that the *E. coli* homolog of YfmR, Uup, plays a direct role in resolving ribosome stalling at polyprolines (114). This observation is consistent with the fact that YfmR and Uup are functionally interchangeable in *B. subtilis*, as Uup can complement the fitness defect of ∆*efp*∆*yfmR* cells (113).

**Fig 5:**
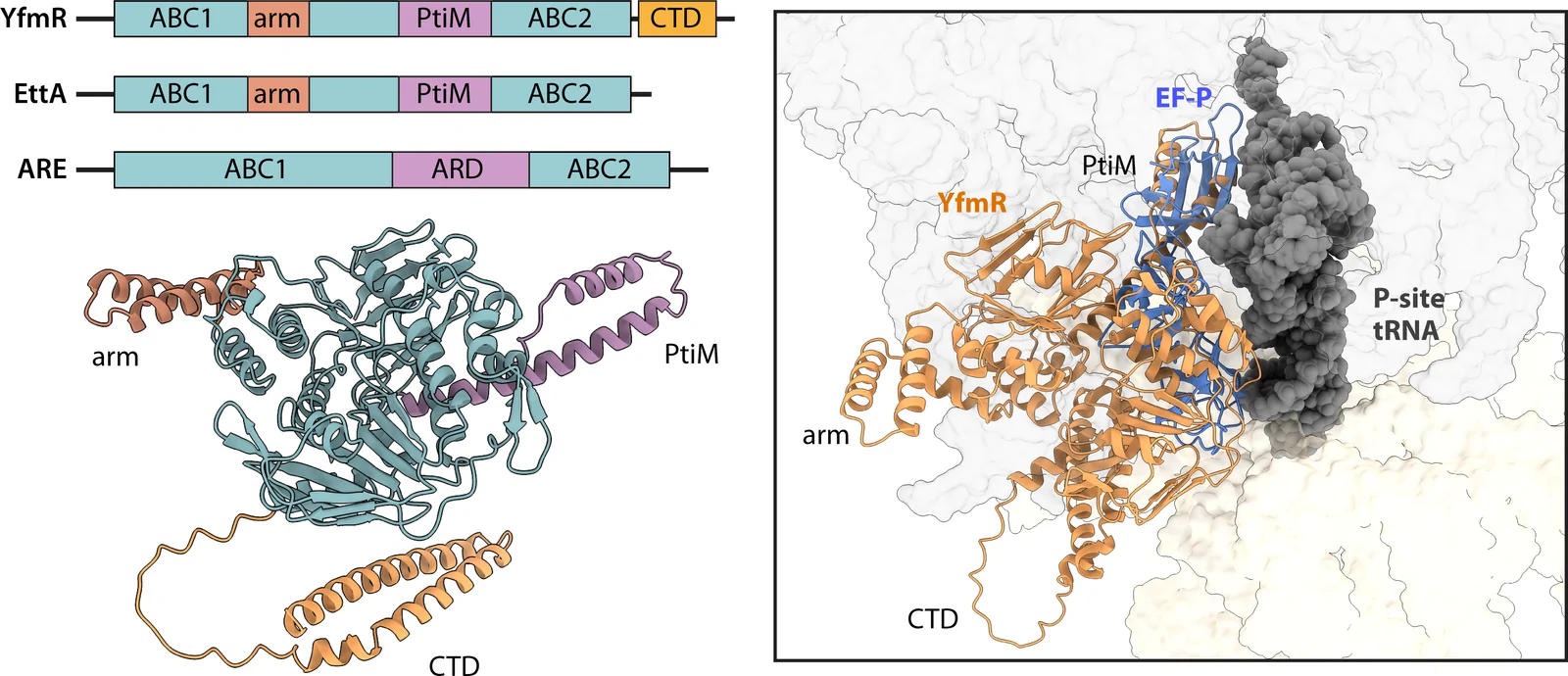
Domain architecture of ABCF ATPases. (Left) ABCFs contain two ATPase domains at the core of the protein. A linker region between the two ABC cassettes extends toward the PTC or even to the exit tunnel in the case of ARE ABCFs. In housekeeping ABCFs, this linker is referred to as the PtIM. In AREs, this domain is typically elongated and is referred to as the antibiotic resistance determinant (ARD). AREs often lack the arm domain. Some ABCFs, like YfmR/Uup, encode a C-terminal extension (CTD) whose function is unknown. (Right) YfmR overlaid on the structure of EF-P bound to a ribosome stalled on a polyproline tract (PDB 6ENU). EF-P and the PtIM of YfmR make similar contacts with the P-site tRNA.

YfmR/Uup is an ABCF ATPase. ABCFs were present in the last universal common ancestor, and there are 45 distinct subfamilies of ABCFs throughout bacteria and eukaryotes (115). An average of 4 unique paralogs are present per bacterial genome, with up to 11 different paralogs in Actinobacteriota and Firmicutes (115). ABCFs in bacteria were first identified and characterized for their role in antibiotic resistance, and this distinct subfamily of ABCFs is classified as antibiotic resistance element (ARE) ABCFs. Since many ABC ATPases are membrane-associated efflux pumps, it was originally hypothesized that antibiotic resistance was conferred by way of efflux. Subsequent studies found that ABCFs lack a transmembrane domain and instead interact with the ribosome to confer target protection (116–119). ARE ABCFs confer resistance to antibiotics that bind the 50S ribosomal subunit and can be distinguished from housekeeping ABCFs by their extended inter-domain linker, termed the antibiotic resistance determinant (ARD), which is analogous to the PtIM of housekeeping ABCFs (Fig. 5). This domain can reach into the PTC, or even into the exit tunnel for some ARE ABCFs (120). Binding of ARE ABCFs causes conformational changes in the ribosome and in the acceptor stem of the P-site tRNA to eject the antibiotic from the ribosome (120–123).

The roles of the housekeeping ABCFs are more mysterious than ARE ABCFs. Like ARE ABCFs, all housekeeping ABCFs investigated to date bind the ribosomal E site (124, 125). The first bacterial housekeeping ABCF to be characterized was EttA (126, 127). EttA binds 70S initiation complex and gates entry into elongation, potentially coordinating protein synthesis with the energy status of the cell. Recent work by Ousalem and colleagues (128) demonstrates that EttA can also work on elongating ribosomes, facilitating translation of acidic residues occurring within the first 30 amino acids of the peptide. This work also noted the similarities between EttA and EF-P in their interactions with the ribosome, as both proteins exhibit tRNA mimicry, interact with the peptidyl-tRNA, and have a domain that reaches toward the PTC. However, EttA is not functionally interchangeable with YfmR *in vivo*, suggesting that it differs in function from YfmR (113). These differences highlight how ABCF paralogs have evolved specialized functions and suggest that there is far more work to be done to determine the roles of uncharacterized ABCFs.

Single deletion of *yfmR*/*uup* does not cause detectable defects in fitness (79, 105, 113). A severe reduction in fitness is only detected in combination with *efp* deletion, indicating that EF-P is still the main player in resolving ribosome stalling. While Hong and colleagues found that *yfmR* deletion from ∆*efp::mls^R^* cells is lethal in *B. subtilis* (113), Takada and colleagues demonstrated that *yfmR* can be deleted from markerless ∆*efp* cells, albeit with a severe synthetic growth defect (105). The *mls* resistance cassette used as a selectable marker in *B. subtilis* imparts resistance to macrolides by methylating the 23S rRNA in the ribosomal exit tunnel, which blocks the antibiotic from binding (129–131). Therefore, lethality of ∆*efp*∆*yfmR* in the presence of the *mls* resistance cassette further implicates the exit tunnel in impeding elongation of polyproline tracts *in vivo*. There is also a synthetic interaction between Uup and EF-P in *E. coli*, as ∆*efp*∆*uup* cells showed a severe fitness defect (79, 114) and a ∆*efp*∆*uup*∆*efpL* strain could not be constructed, suggesting that deletion of all three genes is lethal (79). The importance of maintaining at least one factor to aid polyproline translation highlights the need to translate these sequences and is in line with the presence of polyproline tracts being important for the function of several essential proteins (40).

## THE ROLE OF ABCF NTPase ACTIVITY

The precise role of nucleotide hydrolysis in the function of anti-stalling ABCFs is presently unclear, although both ATP binding and hydrolysis are likely required for activity. ABCFs are classically described as ATPases, although they are more accurately described as NTPases. The eukaryotic ABCF eEF3 efficiently binds and hydrolyzes GTP (132), and the ARE ABCF VgaA functions equally well with ATP, GTP, CTP, or UTP (133). Similarly, EttA is reported to work equally well with ATP vs GTP (126). However, since ATP is present at the highest concentrations *in vivo* (134), ATP is likely the relevant nucleotide. Regardless of nucleotide identity, the role of hydrolysis remains a mystery. EQ2 mutants of ABCF proteins, which contain glutamic acid to glutamine substitutions in the ATP hydrolysis domains of the two ATPase cassettes, are able to bind ATP but not hydrolyze it. These mutants have higher affinity for the ribosome, which suggests that ATP hydrolysis may enable release from the ribosome (124, 125). However, a role of ATP hydrolysis in the catalytic function of the ABCFs remains to be determined.

EttA has been proposed to regulate translation depending on ATP/ADP ratios and thus serve as a hibernation factor and reporter of cellular energy status. However, ATP/ADP ratios are extremely stable, even during starvation (134–136). In contrast, GTP levels are more sensitive to stressors, such as amino acid starvation, and treatment with antibiotics, such as mupirocin and mycophenolic acid (134, 137, 138). Canonical translation factors are GTPases that cannot use ATP. Therefore, one exciting possibility is that, while translational GTPases might become less active when GTP levels are depleted, the ability to use ATP could maintain the functionality of the ABCF ATPases. Moreover, unlike translational GTPases, which are targeted by the alarmones (p)ppGpp, ABCFs may not be targeted by alarmones. This has been shown for the ARE VgaA, where its activity was not inhibited even in the presence of 0.5 mM ppGpp (133). The ability to use ATP and resistance to alarmones likely have physiological importance but have not yet been shown *in vivo*.

## YebC2 AND TACO1 FACILITATE POLYPROLINE TRANSLATION IN BACTERIA AND MITOCHONDRIA

In 2019, Hummels and Kearns (77) performed a screen for suppressors of the motility defect exhibited by ∆*efp* cells in *B. subtilis*. Genes involved in swarming motility in *B. subtilis* are abundant in diproline motifs, most notably in the gene encoding FliY, a component of the flagellar C ring rotor (107). One class of motility phenotype suppressors contained “promoter up” mutations that brought the promoter sequence of a gene called *yeeI* closer to the preferred Sigma A consensus sequence and therefore presumably increased YeeI expression. Although YeeI is annotated as a transcription factor, this finding suggested that increased YeeI expression could facilitate the expression of the diproline-containing FliY flagellar component and therefore might be involved in polyproline translation. The authors further noted that YeeI is homologous to the mitochondrion-localized protein TACO1, which is important for the expression of the proline-rich CoxI protein, again suggesting a possible role in translation (139).

**Fig 6:**
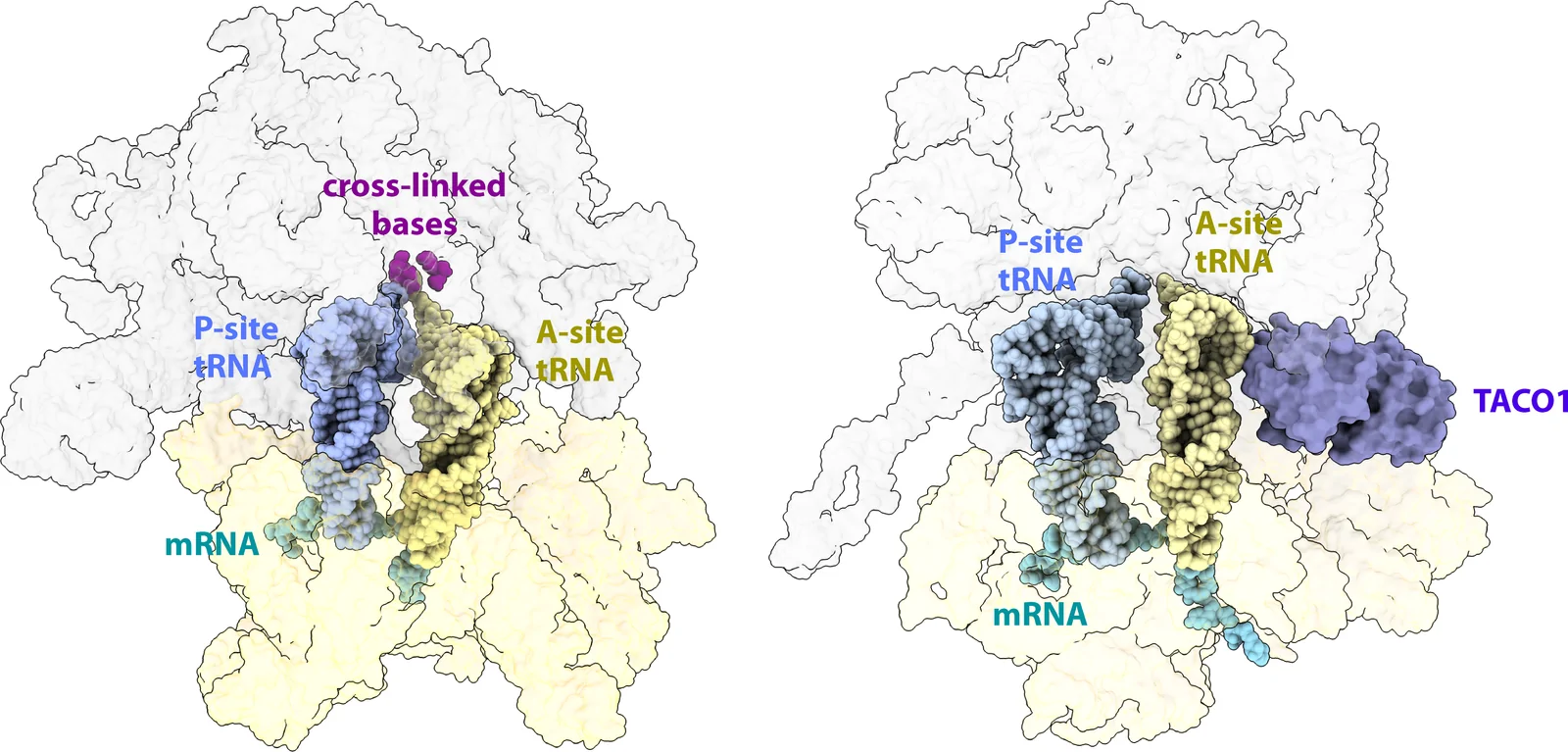
YebC2 and TACO1 interactions with the ribosome. (Left) Site of 23S rRNA bases that cross-linked with YebC2 from *S. pyogenes*. Cross-linking occurred at the PTC, suggesting that YebC2 contacts the PTC when it interacts with the bacterial ribosome. Cross-linking sites are modeled onto a structure of the elongating bacterial ribosome PDB 1VY4. (Right) TACO1 interacts with the ribosomal A site and may stabilize the A-site tRNA. The model was created using 9OLF, the structure of TACO1 bound to the mitoribosome.

YeeI, renamed YebC2, has now been demonstrated to be a ribosome-interacting factor that facilitates translation of polyproline tracts (140, 141). In *Streptococcus pyogenes*, YebC2 was identified as a ribosome-interacting protein through an *in vivo* screen for novel RNA-binding proteins, while in *B. subtilis*, YebC2 was identified in a Tn-seq screen for genes that are important for fitness in the absence of *efp* (113, 141). Reverse transcription mapping of RNA-interacting sites indicates that YebC2 likely interacts with 23S rRNA at the base of helix 89 near the PTC, supporting a direct role for YebC2 in facilitating peptide bond formation (140) (Fig. 6). Ribosome profiling indicates that cells lacking YebC2 exhibit increased ribosome stalling at polyproline motifs, most notably in the conserved tri-proline motif of ValS. Targeted *in vivo* reporters in both *S. pyogenes* and *B. subtilis* further show that loss of YebC2 increases ribosome stalling at a polyproline tract. Finally, YebC2 co-migrates with ribosomes in sucrose density gradients, including with 70S monosomes (113). Altogether, these data strongly support a direct role for YebC2 in translation.

∆*efp*∆*yebC2* cells exhibit a severe fitness defect in *B. subtilis*, suggesting that EF-P and YebC2 have overlapping substrates whose translation is important for growth. The Tn-seq screen that uncovered the genetic interaction between *yfmR* and *efp* also indicated that *yebC2* (*yeeI*) is important for fitness in the absence of *efp* (113). However, ∆*efp*∆*yebC2* cells are not as compromised in fitness as ∆*efp*∆*yfmR* cells, suggesting a greater overlap in substrates between EF-P and YfmR than between EF-P and YebC2 (141). As expected, depletion of EF-P from ∆*yebC2*∆*yfmR* cells is significantly more severe than depletion from either single deletion. These results indicate that each of these three factors acts independently from one another to facilitate translation and that loss of all three factors greatly reduces viability.

The eukaryotic homolog of YebC2 is TACO1, which is imported into mitochondria. Loss of TACO1 causes ribosome stalling on mRNA encoding the cytochrome c oxidase subunits COX1 and COX3 and therefore failure to produce sufficient quantities of these proteins (142). Cytochrome c oxidase is the last member of the electron transport chain and transfers electrons onto oxygen. Therefore, mutations in TACO1 have devastating consequences and cause severe mitochondrial dysfunction and juvenile onset of Leigh Syndrome, which is fatal (142, 143). In 2024, the Rorbach and Barrientos labs showed by Ribo-seq that failure to produce CoxI is due to severe ribosome stalling at polyproline tracts in the CoxI protein transcript (139).

A recent structure of TACO1 bound to the mitochondrial ribosome gives clues about its mechanism of action (144). TACO1 binds to the ribosomal A site in a position that overlaps with the binding site of EF-Tu. Binding of TACO1 increased the structural resolution of the CCA stem of the A-site tRNA, suggesting it promotes tRNA accommodation or may stabilize the A-site tRNA. These data are consistent with a previously proposed model in which TACO1 promotes tRNA accommodation since the fitness of cells lacking TACO1 is further reduced by mutations in bL27m (139). However, direct interaction between TACO1 and bL27m has not been detected by proximity labeling (139) or by structural analysis (144). Alternatively, it has been proposed that TACO1 aids in the release of EF-Tu after tRNA delivery (144). While TACO1 may aid EF-Tu release, this is unlikely to occur through direct competition since TACO1 interacts transiently with the ribosome, and EF-Tu release from the ribosome is governed by GTP hydrolysis on EF-Tu (145, 146).

*In vivo* RNA-protein cross-linking data reveal that YebC2 likely interacts directly with the PTC (140). However, the cryo-EM structure of TACO1 does not reveal a similar site of interaction (Fig. 6) (144). These findings suggest either that YebC2 and TACO1 differ in their interaction with the ribosome or that interaction with the PTC is a transient state that has not yet been resolved by cryo-EM. In support of the latter hypothesis, recent work using small-angle X-ray scattering revealed both open and closed conformations for TACO1 in solution, with domain I being flexible relative to the other two domains (147). Sites on YebC2 that cross-linked with RNA were found in both domains I and II of the protein, and residues in domain I are important for function (140, 141). Since the cryo-EM structure of human TACO1 on the mitoribosome shows TACO1 in the closed conformation, further work is needed to determine whether TACO1 samples additional conformations on the ribosome.

## BEYOND POLYPROLINE TRANSLATION

What additional roles could anti-stalling factors play in translation? The eukaryotic homolog of EF-P (eIF5A) is an extremely versatile translation factor, acting throughout protein synthesis. eIF5A promotes the first cycle of elongation, translation elongation at polyproline tracts and other difficult-to-translate sequences, and translation termination (84, 86, 148–151). EF-P has also been proposed to promote formation of the initial peptide bond, and it is notable that both tRNA^fMet^ and tRNA^Pro^ are the only tRNAs to contain the CpU-bulge caused by a mismatch in the D-loop of the tRNA that contacts EF-P and could therefore impart specificity. Interestingly, single molecule tracking experiments in live cells indicate that EF-P interacts transiently with the ribosome throughout the course of elongation, but only tRNA^fMet^ and tRNA^Pro^ in the P site promote stable binding (152). An additional piece of evidence for EF-P function in early elongation is that there are greater levels of initiator tRNA^fMet^ associated with ribosomes harvested from cells lacking EF-P and YfmR (113). However, FMT, which is responsible for formylating methionine on initiator tRNA^fMet^, has a polyproline tract, and so the increased association of initiator tRNAs with ribosomes of cells lacking EF-P and YfmR may be due to reduced expression of FMT. While it is plausible that EF-P could promote early elongation, there are notable differences between EF-P and eIF5A, with eIF5A lacking the domain that interacts with the E-site codon (51). Most crucially, evidence for a role in early elongation has not yet been suggested by ribosome profiling experiments.

YfmR alleviates ribosome stalling at proline-rich motifs, but thus far, only a small number of *in vivo* reporters have been tested (105, 113, 114). Polyproline reporters were a logical first step, given the severe fitness defects associated with ∆*efp*∆*yfmR* cells. However, YfmR is likely to interact with other substrates given its strong association with actively translating ribosomes (113). In support of this hypothesis, Katoh and Suga (153) recently showed that Uup/YfmR increases incorporation rates of consecutive non-canonical amino acids. In these assays, noncanonical amino acids were charged to a chimeric tRNA containing the D arm of tRNA^Pro^. However, Uup/YfmR could enhance amino acid incorporation even when the charged tRNA was a native tRNA, suggesting that Uup/YfmR may have less tRNA specificity than EF-P. These results suggest that ABCFs may also have uses in the field of synthetic biology, where they could be employed to enhance production of proteins that do not exist in nature.

YebC2 is a member of the YebC protein family, members of which have been characterized as transcription factors (154–157). However, YebC2 clades separately from the YebC transcription factor proteins. Notably, many bacteria encode both a YebC and a YebC2 protein, which is also consistent with the model that these proteins have divergent functions. YebC-family proteins that are known to play a role in transcription tend to clade with YebC, while those that play a role in translation tend to clade with YebC2. However, there are exceptions to this rule. For example, there is evidence that YebC from *E. coli* functions in both transcription and translation (140). *In vitro*, *E. coli* YebC exhibited greater rescue of ribosome stalling than YebC2 (YeeN). Additionally, in *Salmonella typhimurium*, ∆*efp*∆*yebC2* cells did not exhibit a growth defect, whereas *yebC* cannot be deleted from the ∆*efp* background, suggesting that YebC, rather than YebC2, has overlapping function with EF-P in *S. typhimurium* (140). Finally, YebC2 from *S. pyogenes* is important for expression of the virulence factor SpeB. Differential expression was only dependent on the promoter and not the open reading frame, suggesting that these differences are due to transcription and not translation (140).

Since *E. coli* YebC and *S. pyogenes* YebC2 both demonstrate the potential for function in transcription and translation, it raises the possibility that other YebC-family proteins have a dual role as well. This is not entirely unexpected, since RNA helices present in the ribosome and in the acceptor stem of tRNA are somewhat similar to a DNA helix. However, evidence for the role of YebC-family proteins promoting transcription is indirect in most cases. Most support for YebC function in transcription is based on differences in mRNA transcript levels. These differences are usually in a very small (<10) subset of mRNAs and could be due to differential translation rather than differential transcription, since decreased translation results in a lack of ribosomal protection that can cause mRNA degradation. Moreover, ribosome stalling can also lead to mRNA cleavage and reduced transcript levels. To date, the most direct evidence for a transcriptional role is from *Pseudomonas aeruginosa*, where YebC negatively regulates the quorum-sensing response regulator *pqsR* (154). Deletion of *yebC* caused increased expression of *pqsR* and YebC bound to the *pqsR* promoter in an electrophoretic mobility shift assay. More direct biochemical studies of diverse YebC-family proteins are needed to further clarify the precise roles of these proteins in both transcription and translation.

## OUTLOOK

Ribosomes use a broad range of substrates to give rise to the diverse host of proteins that exist in nature. Such a feat is unique in the world of enzymes because, although the peptide bond is always the same, the molecules at either end of this bond are highly variable. Ribosomes can also form peptide bonds between non-canonical and man-made amino acids, which has important implications for synthetic biology. While the catalytic activity belongs to the ribosome, peptide bond formation is greatly aided by accessory translation factors such as EF-P, YfmR, and YebC2.

Recently, the role of ABCFs in facilitating translation has brought this class of proteins into greater focus (105, 113, 114, 128, 158). Nevertheless, our mechanistic understanding of these factors is still in its infancy, especially given the diversity of these proteins. Cryo-EM structures of several housekeeping ABCFs reveal a consistent theme in which ABCF-facilitated movement of the acceptor stem of the P-site tRNA may prime the ribosome for A-site tRNA accommodation (124, 127, 159). Alternatively, ABCFs may promote peptide bond formation by stabilizing the peptidyl-tRNA in a manner similar to EF-P (51). While current structures lay a necessary foundation, these ABCF-ribosome complexes have been assembled *in vitro* using 70S initiation complexes or with 70S elongation complexes using ATPase-defective (EQ2) versions of the ABCFs. Therefore, an important direction for future work will be to determine the structures of ABCFs on their preferred substrates. Moreover, further biochemical and structural work will reveal the precise function of ABCF NTPase activity, the role of C-terminal extensions that vary in length, and precise interactions between residues in the PtIM and the P-site tRNA.

Another avenue for future research is to determine roles for anti-stalling factors outside of translation elongation. Recently, a comprehensive cryo-EM study of the stages of CAT tailing in eukaryotes revealed an essential role of eIF5A in this process (160). CAT-tailing is part of the RQC pathway and results in the addition of Ala and Thr residues to the C-terminus of aberrant peptides and targets them for degradation (161–165). During RQC in eukaryotes, the small and large ribosomal subunits of stalled/collided ribosomes are dissociated, leaving the large subunit obstructed by peptidyl-tRNA. Rqc2 then delivers Ala- or Thr-charged tRNAs to the large subunit in the absence of mRNA template, the ribosomal small subunit, and elongation factors. eIF5A binds to the E site even before the recruitment of Rqc2-bound tRNA. Once the A-site tRNA has been accommodated with the help of Rqc2, eIF5A then aids peptidyl transfer from the P-site tRNA to the A-site tRNA. The RQC pathway in bacteria is similar, with RqcH performing the role of Rqc2 to help clear obstructed ribosomes that are the product of ribosome collisions and ribosome splitting (60, 166, 167). RqcH is aided by RqcP or YlmH (168), small proteins that likely serve the role of eIF5A. However, not all bacteria that rely on RQC encode RqcP or YlmH (168), raising the possibility that EF-P could serve this role in these organisms.

EF-P, YebC-family proteins, and ABCFs are present in bacteria, archaea, and eukaryotes. As these proteins have co-evolved with the species that harbor them, a better understanding of the evolutionary history of these proteins is likely to yield crucial insight into their diverse functions. For example, a comprehensive survey of the evolution and diversification of ABCFs recently helped to uncover new potential families of AREs in antibiotic-producing bacteria (115). This same study also identified ABCFs that likely arose in eukaryotes through endosymbiotic gene transfer. TACO1 likely also arose through an endosymbiotic gene transfer event since it is more similar in sequence to YebC2 than to YebC, whereas archaea that are closely related to the ancestor of eukaryotes encode a protein that has more sequence similarity to YebC (169). Altogether, the widespread conservation of factors like EF-P, YebC2, and the ABCFs indicates that they are important in essentially every environment that sustains life.
